# Retraction Note: Sedum sarmentosum Bunge extract ameliorates lipopolysaccharide- and D-galactosamine-induced acute liver injury by attenuating the hedgehog signaling pathway via regulation of miR-124 expression

**DOI:** 10.1186/s12906-022-03796-7

**Published:** 2022-11-22

**Authors:** Li Hao, Ming-wei Liu, Song-tao Gu, Xue Huang, Hong Deng, Xu Wang

**Affiliations:** 1 Department of Emergency, Yan’an Hospital of Kunming City, Panlong District, 245 Renmin East Road, Kunming, 650051 China; 2 Department of Emergency, First Affiliated Hospital of Kunming Medical University, 295 Xichang Road, Wu Hua District, Kunming, 650032 China

**Retraction Note: BMC Complement Med Ther 20, 88 (2020)**

**https://doi.org/10.1186/s12906-020-2873-1**

The Editor has retracted this article because of overlap within figures, specifically:

- Figure 6A (panel “Hedgehog”) overlaps with Figure 6A (panel “Gli”)
- Figure 11A (panel “Model SSB group”) overlaps with Figure 11A (panel “Model Silymarin group”)

The Editor therefore no longer has confidence in the integrity of the data.

Xu Wang does not agree with this retraction. None of the other authors has responded to any correspondence from the Editor about this retraction.

